# Supplementary material for: Binding moral values gain importance in the presence of close others
Source: Nat Commun. 2021 May 11;12:2718. doi: 10.1038/s41467-021-22566-6 (PMC8113481; doi:10.1038/s41467-021-22566-6)
Supplement: Supplementary file 1 — Supplementary Information [file 41467_2021_22566_MOESM1_ESM.pdf]

Binding Moral Values Gain Importance in the Presence of Close Others

Daniel A. Yudkin[1], Ana Gantman[2], Wilhelm Hofmann[3], & Jordi Quoidbach[4]

Supplementary Online Materials

[1] Social and Behavioral Science Initiative, University of Pennsylvania, PA
[2] Brooklyn College, New York
[3] Ruhr University Bochum, Germany
[4] ESADE Business School, Spain

Study 1

1.1. Affective state

One important consideration that arises in the context of the primary analyses is the effect of participants' current affective state. It is possible, for example, that being in the presence of close others may increase people's happiness, which subsequently increases the importance they place on moral values. Normally, in order to address this issue we would simply include affective state as a covariate in the regression analyses. However, more than 25% of the timepoints did not include assessments of affective state. Thus including affective state in the analyses significantly cuts down on the statistical power available to detect these (small) effects by considerably diminishing the sample size.

In order to take into account the effects of current affective state without creating unacceptable reductions in sample size, we used an imputation procedure[1]. The imputation procedure was implemented using the *mice* package in R[2], which is based on fully conditional specification. We used a predictive mean matching method, which reduces bias by drawing real values sampled from the data. The model was set at 20 iterations. The algorithm then runs the prespecified regression model on each dataset separately and pools the results.

Results of the imputation procedure show a significant main effect of relationship type on moral importance, $F(4, 1{,}616) = 6.45$, $p < .001$. Furthermore, as predicted, the importance of binding values showed a significant relationship with social closeness, $B = .040$, $SE = .011$, $t(2{,}225) = 3.46$, $p < .001$, as did individualizing values, $B = .028$, $SE = .009$, $t(2{,}350) = 2.98$, $p = .002$. The difference between these slopes was marginally significant, $B = .029$, $SE = .17$, $t(1{,}109) = 1.76$ $p = .078$. Thus, while the interaction effect drops to marginal significance in the imputed dataset, we still find that the importance of binding values is positively associated with social closeness.

1.2. Interpretation of effect sizes

It can be difficult to interpret the effect size of a change in the ratings of moral importance in absolute terms. One way to interpret the effect sizes is to examine the impact of social relationships on moral importance relative to the typical range of people's fluctuations in moral importance ratings (i.e., the difference between the maximum and minimum importance value reported by an individual). Indeed, if an individual reports moral importance ratings that mostly vary from 75 to 90, a change of 3 points corresponds to 20% of the range in moral importance,

which is substantial. Conversely, if their moral importance ratings vary across the range from 0 to 100, then a change of 3 points would be negligible. The median range for the importance of binding values was 14.5; as such, the effect of being with one's best friend versus with an acquaintance on binding moral values ($\simeq$ 4.0 points) represents a 27.5% change in participants' normal range, which is far from trivial.

Another way of thinking about the effect sizes is to compare them to other well-known changes in moral evaluation. For example, it is well-established in literature on morality that moral values change over the course of the lifespan. In particular, other work shows that every ten years increase in age is associated with approximately a .19 SD increase in the importance of binding moral values[3]. In comparison, we find that, relative to being with an acquaintance, being with a best friend is associated with an expected .093 SD increase in the importance of binding values (49% of the effect size of a decade increase in age). In other words, being in the presence of an acquaintance versus best friend causes a shift in the importance of binding values equivalent to being about 5 years older.

1.3. Relationship frequencies

Supplementary Table 1. Frequency of occurrence of each relationship type. The right column indicates the number of responses in Study 1 in which people indicated they were in the presence of that person.

Relationship Type	Frequency
Alone	806
Acquaintance	43
Best Friend	19
Brother	28
Child	215
Client	13
Colleague/Classmate	138
Extended Family	62
Father	50
Friend	68
Mother	84
Romantic Partner	316
Sister	31
Stranger	78

1.4. Individual value breakdown

While the primary analysis in the main paper looks at different effects of social closeness on the averaged value of binding versus individualizing values, we can also look at the effects broken down by each value separately. The figure below shows this analysis for each value. There appears to be a significant or marginally significant effect of closeness on every value except harm, which shows no relationship.

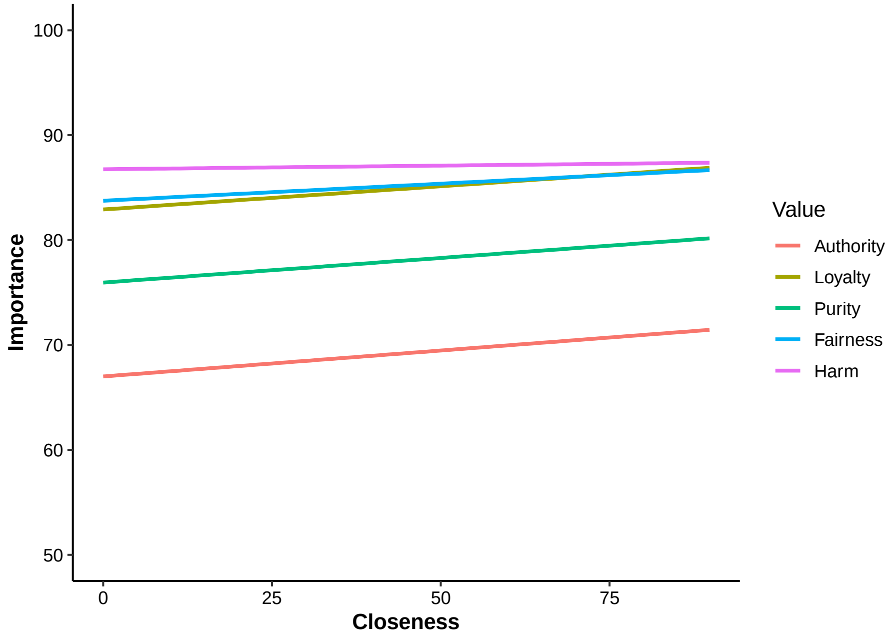

Supplementary Figure 1 The relationship between social closeness according to each of the separate moral foundations measured in Study 1 (n = 1,166 participants). The values of each of the slopes, controlling for time of day, day of week, age, and gender, are as follows: authority, B = 0.05, SE = 0.02, t(1029) = 2.83, p = 0.005; loyalty, B = 0.05, SE = 0.03, t(266) = 1.87, p = 0.063; purity, B = 0.04, SE = 0.02, t(867) = 2.30, p = 0.021; fairness, B = 0.03, SE = 0.02, t(598) = 1.99, p = 0.047; harm, B = 0.01, SE = 0.01, t(1041) = 0.57, p = 0.566. Ribbons indicate *SEM*.

1.5. User interface

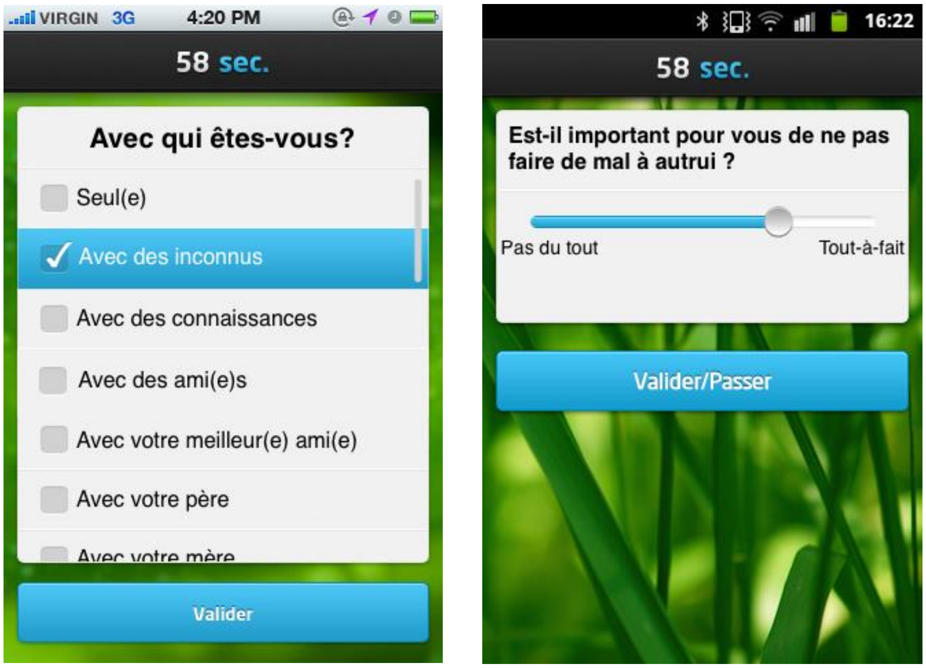

Supplementary Figure 2. Screenshots of the "58 Seconds" experience sampling project assessing social context and moral importance (*n* = 1,166 participants). In the left panel (social context), the heading is translated as "Whom are you with?", followed by a variety of options including "alone", "with strangers," "with acquaintances," etc. In the right panel (moral importance), the question is translated as, "How important is it to you to avoid doing harm to others?" (Not at all; Completely). Participants were asked whom they were with at each timepoint; moral importance items were randomly administered across timepoints from a larger subset of questions.

Study 2

2.1. Breakdown by individual foundation

Here we show the relationship of each moral value with estimated closeness.

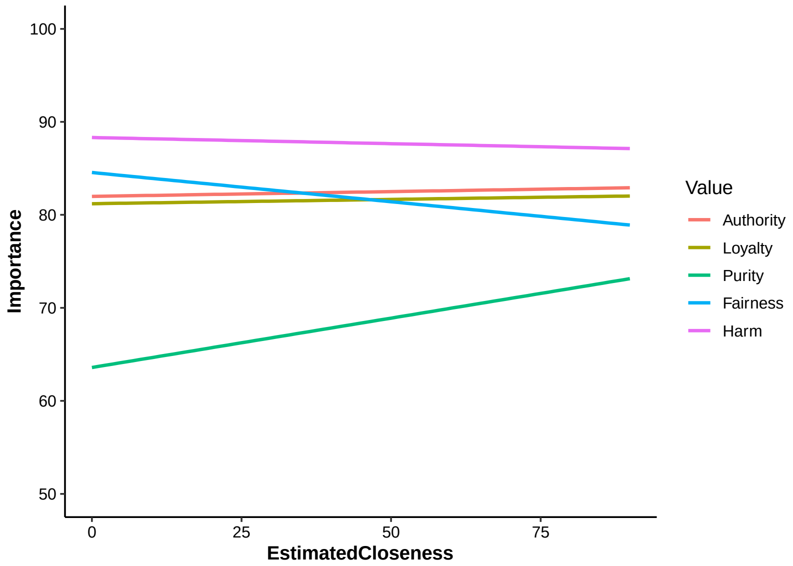

Supplementary Figure 3. The relationship between estimated social closeness (*n* = 110 participants) and the importance of each moral value (*n* = 2,016 participants, Study 2). The values of each of the slopes, controlling for age, gender, mood and political orientation, are as follows: authority, *B* = -0.01, *SE* = 0.01, *t*(2716) = -1.43, *p* = 0.152; loyalty, *B* = -0.01, *SE* = 0.01, *t*(2718) = -0.86, *p* = 0.391; purity, *B* = 0.04, *SE* = 0.01, *t*(2712) = 2.90, *p* = 0.004; fairness, *B* = -0.05, *SE* = 0.01, *t*(2718) = -5.28, *p* < 0.001; harm, *B* = -0.02, *SE* = 0.01, *t*(2717) = -2.73, *p* = 0.006. Ribbons indicate *SEM*

Study 3

3.1 Breakdown by individual foundation.

Here we present the effects of condition according to each individual moral value in Study 3.

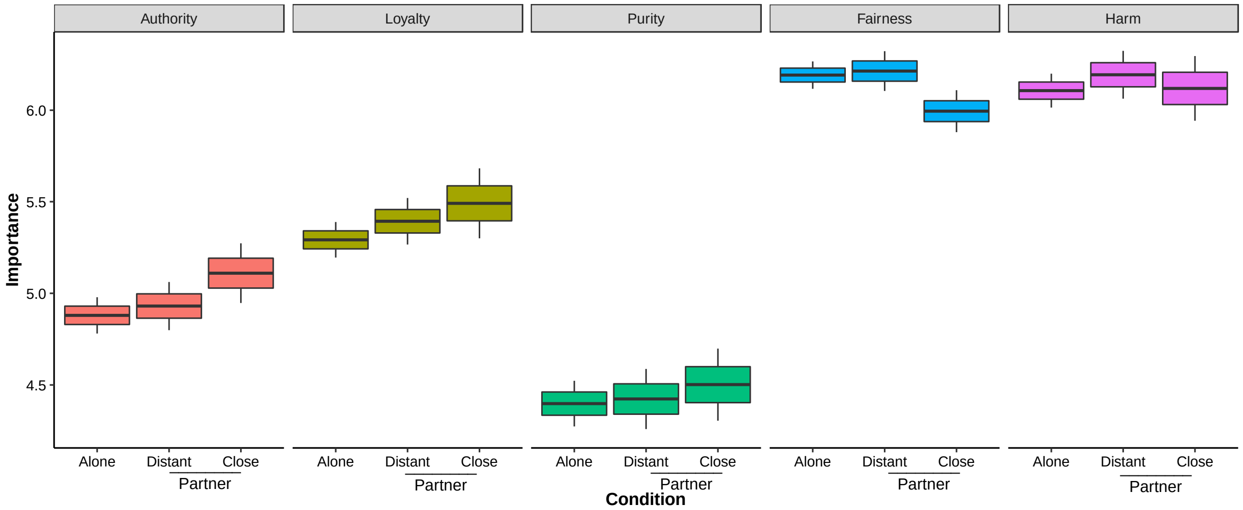

Supplementary Figure 4. Post-hoc assigned condition (alone, partner distant, partner close) on the importance assigned to each moral value as measured by the Moral Foundations Questionnaire (n = 390 participants) in Study 3. Differences between means are significant at the following levels for each value, respectively: authority, $F(2, 340) = 3.92$, $p = 0.021$; loyalty, $F(2, 340) = 3.71$, $p = 0.026$; purity, $F(2, 340) = 0.64$, $p = 0.526$; fairness, $F(2, 340) = 3.43$, $p = 0.034$; harm, $F(2, 340) = 0.80$, $p = 0.452$. Box centers reflect group means; edges *SEM*; whiskers 95% CI.

Studies 4A-C

4.1 Correlational Analysis

Here we present the statistics of the correlational analysis examining the relationship between self-rated closeness and binding versus individualizing moral importance in Study 4A. The interaction was significant at *B* = -0.08, *SE* = 0.02, $t(1930) = -5.00$, $p < 0.001$.

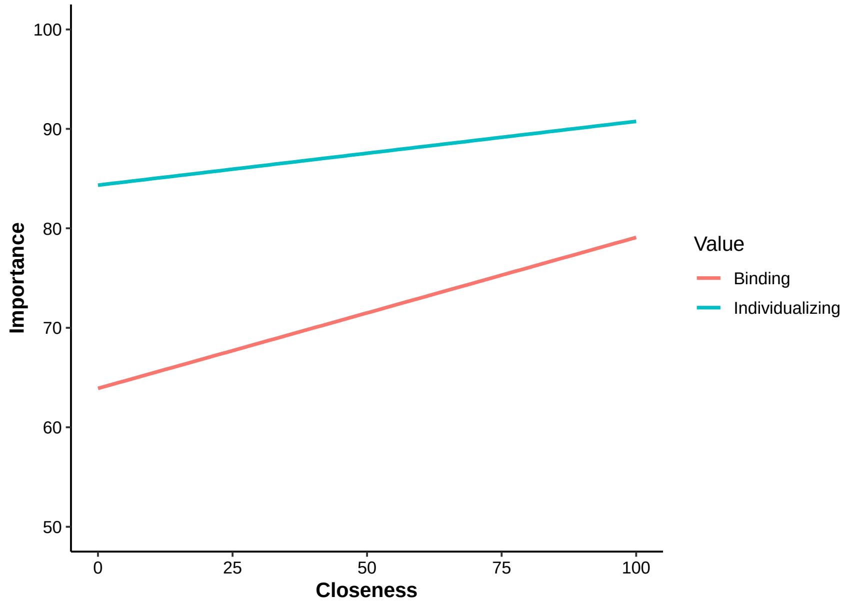

Supplementary Figure 5. Relationship between self-rated closeness of whom participants are with at the moment, and the importance of binding versus individualizing values (Study 4A, *n* = 2,031 participants). Ribbons *SEM*.

4.2 Individual Value Breakdown

Here we present the effects of condition according to each individual moral value in Study 4B.

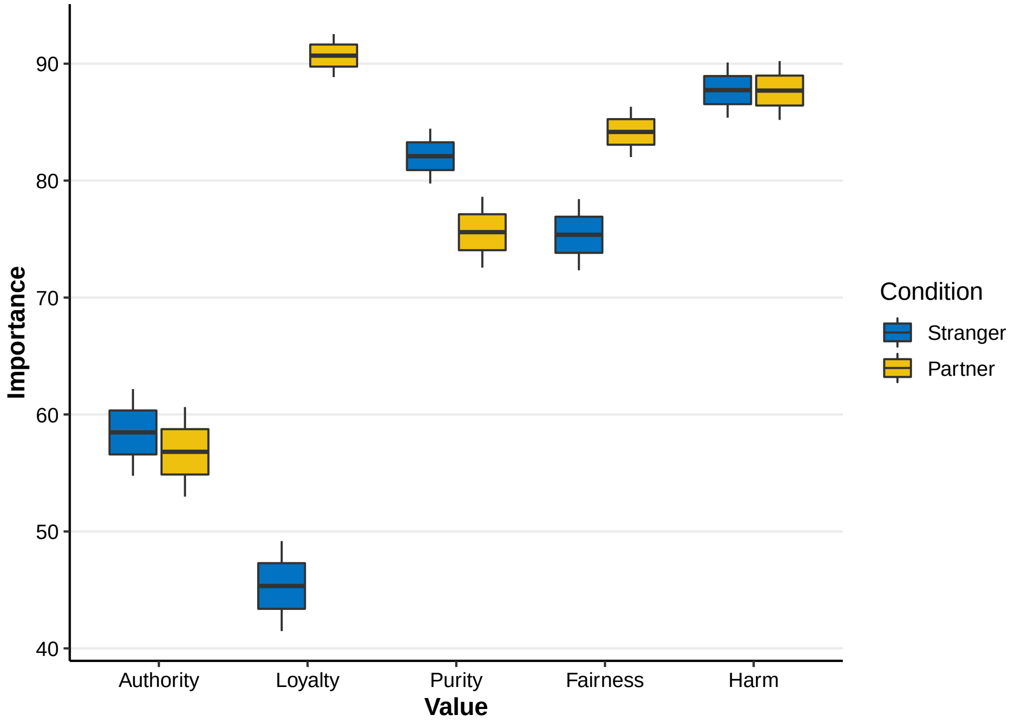

Supplementary Figure 6. Relative importance of each value in the "romantic partner" versus "stranger" condition in Study 4B, (n = 580 participants). Pairwise differences according to condition within each value are as follows: authority, $t(558) = -0.61$, $p = .54$; loyalty, $t(564) = 20.79$, $p < .001$; purity, $t(563) = -3.36$, $p < .001$; fairness, $t(561) = 4.61$, $p < .001$; harm, $t(561) = -0.02$, $p = .98$. Box centers reflect group means; edges *SEM*; whiskers 95% CI

4.3. Individual value breakdown for Study 4C.

Here we present the effects of condition according to each individual moral value in Study 4B.

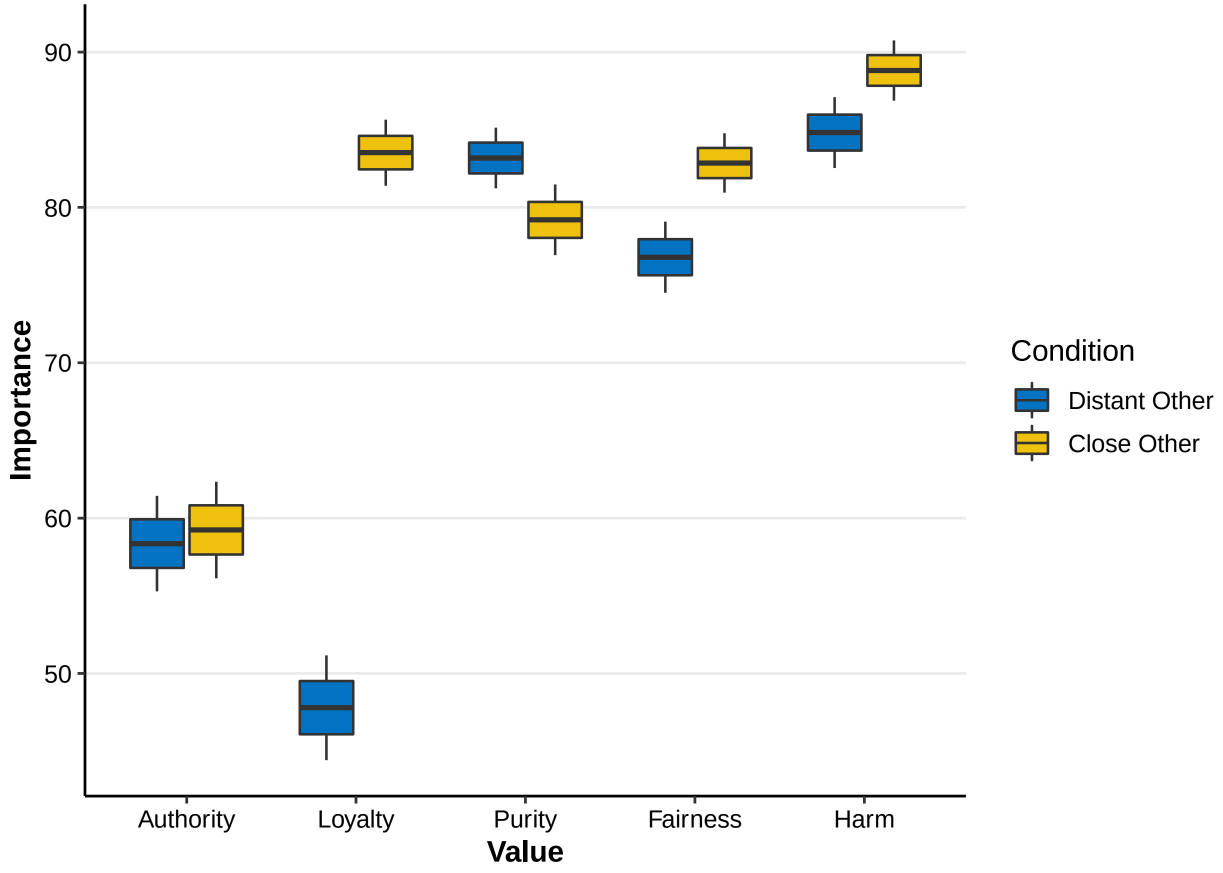

Supplementary Figure 7. Relative importance of each value in the "close other" versus "not close other" condition in Study 4C (n = 752 participants). Pairwise differences according to condition within each value are as follows: authority, $t(728) = 0.39$, $p = .69$; loyalty, $t(734) = 17.64$, $p < .001$; purity, $t(563) = -3.36$, $p < .001$; fairness, $t(733) = -2.61$, $p < .001$; harm, $t(731) = 2.63$, $p = .008$. Box centers reflect group means; edges *SEM*; whiskers 95% CI

SUPPLEMENTARY REFERENCES

[1] Graham, J. W. (2009). Missing data analysis: Making it work in the real world. *Annual review of psychology*, *60*, 549-576.
[2] Stef van Buuren, Karin Groothuis-Oudshoorn (2011). mice: Multivariate Imputation by Chained Equations in R. *Journal of Statistical Software*, 45(3), 1-67.
[3] Hawkins, S., Yudkin, D., Juan-Torres, M., & Dixon, T. (2018). Hidden tribes: A study of America's polarized landscape. *New York: More in Common.*
