# Supplementary material for: Binding moral values gain importance in the presence of close others
Source: Nat Commun. 2021 May 11;12:2718. doi: 10.1038/s41467-021-22566-6 (PMC8113481; doi:10.1038/s41467-021-22566-6)
Supplement: Supplementary file 3 — Reporting Summary [file 41467_2021_22566_MOESM3_ESM.pdf]

nature research

Corresponding author(s): Daniel Yudkin

Last updated by author(s): 2021-2-3

Reporting Summary

Nature Research wishes to improve the reproducibility of the work that we publish. This form provides structure for consistency and transparency in reporting. For further information on Nature Research policies, see our Editorial Policies and the Editorial Policy Checklist.

Statistics

For all statistical analyses, confirm that the following items are present in the figure legend, table legend, main text, or Methods section.

n/a	Confirmed	
☐	☒	The exact sample size (n) for each experimental group/condition, given as a discrete number and unit of measurement
☐	☒	A statement on whether measurements were taken from distinct samples or whether the same sample was measured repeatedly
☐	☒	The statistical test(s) used AND whether they are one- or two-sided<br>*Only common tests should be described solely by name; describe more complex techniques in the Methods section.*
☐	☒	A description of all covariates tested
☐	☒	A description of any assumptions or corrections, such as tests of normality and adjustment for multiple comparisons
☐	☒	A full description of the statistical parameters including central tendency (e.g. means) or other basic estimates (e.g. regression coefficient) AND variation (e.g. standard deviation) or associated estimates of uncertainty (e.g. confidence intervals)
☐	☒	For null hypothesis testing, the test statistic (e.g. F, t, r) with confidence intervals, effect sizes, degrees of freedom and P value noted<br>*Give P values as exact values whenever suitable.*
☐	☒	For Bayesian analysis, information on the choice of priors and Markov chain Monte Carlo settings
☐	☒	For hierarchical and complex designs, identification of the appropriate level for tests and full reporting of outcomes
☐	☒	Estimates of effect sizes (e.g. Cohen's d, Pearson's r), indicating how they were calculated

Our web collection on statistics for biologists contains articles on many of the points above.

Software and code

Policy information about availability of computer code

Data collection	Qualtrics, Versions 2019-2021
Data analysis	R version 4.0.1 (2020-06-06)

For manuscripts utilizing custom algorithms or software that are central to the research but not yet described in published literature, software must be made available to editors and reviewers. We strongly encourage code deposition in a community repository (e.g. GitHub). See the Nature Research guidelines for submitting code & software for further information.

Data

Policy information about availability of data

All manuscripts must include a data availability statement. This statement should provide the following information, where applicable:

- Accession codes, unique identifiers, or web links for publicly available datasets
- A list of figures that have associated raw data
- A description of any restrictions on data availability

The datasets generated for the current study are available in the Open Science Framework repository, https://osf.io/4q8jg/, DOI: 10.17605/OSF.IO/4Q8JG

Field-specific reporting

Please select the one below that is the best fit for your research. If you are not sure, read the appropriate sections before making your selection.

☐ Life sciences ☒ Behavioural & social sciences ☐ Ecological, evolutionary & environmental sciences

For a reference copy of the document with all sections, see nature.com/documents/nr-reporting-summary-flat.pdf

Behavioural & social sciences study design

All studies must disclose on these points even when the disclosure is negative.

Study description

In a quantitative design, participants answered questions about how close they felt to the people they were with and how important moral values were to them.

Research sample

Study 1. The research was part of the "58 seconds" project investigating the everyday life of European adults , approved by The Ethics Committee of ESADE Business School, Spain, approval number 005/2019. Upon signing up for and providing consent to participate in the study, participants (N = 1,166, Mage = 35.7, SD = 11.1, 861 female, 305 male) provided demographic information including their age and gender. They were then contacted at random points over the next several months and asked to respond to a series of questions (pulled from a larger pool).

Study 2, participants. The research was approved by the Human Research Protections Program at the Institutional Review Board, University of Pennsylvania, Protocol #834222. The study was preregistered at https://aspredicted.org/blind.php?x=6sw3tn. No participants were excluded from analysis, leaving a final sample of 2,016 participants (Mage = 36.9, SD = 11.8, 954 male, 1,038 female, 24 other/fluid), who participated through Amazon's Mechanical Turk via TurkPrime for compensation of 20¢. Questions for this and all subsequent surveys were administered through Qualtrics Versions 2019-2021.

Study 3, participants. The research was approved by the Human Research Protections Program at the Institutional Review Board, University of Pennsylvania, Protocol #834222. This study was implemented at the University of Pennsylvania with undergraduates as participants. The overall purpose of the study was to determine whether the importance people afforded moral values depended on whether they were with another person and how close they felt to that person. Thus, we planned to randomly assign participants to either the "alone" or the "partner" condition and then measure people's sense of social closeness to their partner.
Overall, a total of 404 participants ended up registering to participate in the study, advertised as "Who Do You Think You Are?" via an online recruitment tool (SONA), in exchange for course credit. The final sample consisted of 390 participants (Mage = 19.8, SD =1.2, 267 female, 122 male, 1 other/fluid).

Study 4A, participants. The research was approved by the Human Research Protections Program at the Institutional Review Board, University of Pennsylvania, Protocol #834222. The study was preregistered at https://aspredicted.org/blind.php?x=fr9fd3. The final sample consisted of 2,031 (Mage = 32.2, SD = 11.7, 867 male, 1050 female, 26 other/fluid, 88 missing), who participated through Amazon's Mechanical Turk via TurkPrime for compensation of 50¢.

Study 4B, participants. The research was approved by the Human Research Protections Program at the Institutional Review Board, University of Pennsylvania, Protocol #834222. The study was preregistered at https://aspredicted.org/blind.php?x=f9796m. Our power analysis was based on pretesting that suggested an approximate effect size of d = .3. The final sample consisted of 580 participants (Mage = 32.5, SD = 11.5, 211 male, 367 female, 2 other/fluid), who participated through Prolific for compensation of 25¢.

Study 4C, participants. The research was approved by the Human Research Protections Program at the Institutional Review Board, University of Pennsylvania, Protocol #834222. The study was preregistered at https://aspredicted.org/blind.php?x=f9796m. The final sample consisted of 752 participants (Mage = 33.2, SD = 12.5, 272 male, 479 female, 1 other/fluid), who participated through Prolific for compensation of 25¢.

Sampling strategy

Study 1: All participants who agreed to participate in the larger study were included for analysis

Study 2: Our power analysis was based on the overall main effect of the presence-of-others on moral importance in Study 1, with an approximate effect size of d = 0.15 (a very small effect). A power analysis conducted in G*Power suggested a required sample size of 1,870 (power = 90%). To be conservative, we aimed to collect a sample of about 2,000.

Study 3: Because the social context that people were going to be in in the "partner" condition was that of "colleague/classmate," we based our power analysis off the analogous results in Study 1. Probing the difference in moral importance for people in this category (versus alone) yielded an effect size of about d = 0.2 (a small effect). Because the lab-based nature of the study allowed us to reduce additional variance introduced by other contextual differences (e.g., differences in the environment in which the questions were answered), we estimated an effect size of d = 0.3. A power analysis in G*Power suggested a minimum sample size of 278 is required to detect an effect of this size (power = 80%, one-tailed); we sought to oversample in order to maximize our changes of detecting an effect.

Study 4A: Our power analysis was based on pretesting that suggested an approximate effect size of .1 < d < .2. A power analysis conducted in G*Power suggested a required sample size of about 2,000 (power = 90%) would be capable of detecting an effect size of d = .14.

Study 4B: A power analysis conducted in G*Power suggested a required sample size of about 580 (power = 95%); we sought 600 to be conservative.

	Study 4C: For this preregistered study, our power analysis was based on pretesting that suggested an approximate effect size of d = .25. A power analysis conducted in G*Power suggested a required sample size of about 800 (power = 95%).
Data collection	Study 1: Participants responded through their smartphone.<br>Studies 2-5: Data was collected via Qualtrics Version 2020 software. The researcher was present in Study 3 and not blind to condition or hypothesis.
Timing	Study 1: 5/27/13 - 3/12/14<br>Study 2: 11/2019<br>Study 3: 10/2019 - 4/2020<br>Study 4A-4C: 8/2020
Data exclusions	Study 1: No participants were excluded from analysis.<br>Study 2: No participants were excluded from analysis.<br>Study 3: We excluded four participants for failing to follow instructions, four for failing an attention check, and six participants who knew experimenter beforehand, since a relationship between the participant and the experimenter introduced the possibility that a sense of being in a social situation would be activated even among participants who were in the “alone” condition.<br>Study 4A: We only included for analysis participants that passed an attention check (how many fatal heart attacks they had had in their life) were not using a proxy server or VPN, and were in the presence of others.<br>Study 4B: We only included for analysis participants that passed an attention check (how many fatal heart attacks they had had in their life) were not using a proxy server or VPN.<br>Study 4C: We only included for analysis participants that passed an attention check (how many fatal heart attacks they had had in their life) were not using a proxy server or VPN.
Non-participation	No participants dropped out or declined participation.
Randomization	Participants in Experiments 1-2 were not randomly assigned. Participants in Experiment were randomly assigned to an "alone" versus a "partner" condition using a random number generator. Participants in Experiments 4A-C were randomly assigned to a "close" versus "distant" condition using a random number generator.

Reporting for specific materials, systems and methods

We require information from authors about some types of materials, experimental systems and methods used in many studies. Here, indicate whether each material, system or method listed is relevant to your study. If you are not sure if a list item applies to your research, read the appropriate section before selecting a response.

Materials & experimental systems

n/a	Involved in the study
☒	☐ Antibodies
☒	☐ Eukaryotic cell lines
☒	☐ Palaeontology and archaeology
☒	☐ Animals and other organisms
☐	☒ Human research participants
☒	☐ Clinical data
☒	☐ Dual use research of concern

Methods

n/a	Involved in the study
☒	☐ ChIP-seq
☒	☐ Flow cytometry
☒	☐ MRI-based neuroimaging

Human research participants

Policy information about studies involving human research participants

Population characteristics	See above.
Recruitment	Participants in Study 1 were self-selected to participate in the 58 seconds survey. Participants in Study 2, and 4A-C were online workers. Participants in Study 3 were undergraduates at University of Pennsylvania.
Ethics oversight	Study 1: The Ethics Committee of ESADE Business School, Spain. Other studies: Institutional Review Board, University of Pennsylvania

Note that full information on the approval of the study protocol must also be provided in the manuscript.
